# Great apes distinguish true from false beliefs in an interactive helping task

**DOI:** 10.1371/journal.pone.0173793

**Published:** 2017-04-05

**Authors:** David Buttelmann, Frances Buttelmann, Malinda Carpenter, Josep Call, Michael Tomasello

**Affiliations:** 1 Department of Developmental and Comparative Psychology, Max Planck Institute for Evolutionary Anthropology, Deutscher Platz 6, Leipzig, Germany; 2 Department of Developmental Psychology, University of Bern, Fabrikstraße 8, CH Bern, Switzerland; 3 Department of Developmental Psychology, Friedrich-Schiller-University Jena, Am Steiger 3/1, Jena, Germany; 4 School of Psychology & Neuroscience, University of St Andrews, St Mary’s Quad, St Andrews, Scotland, United Kingdom; 5 Department of Psychology and Neuroscience, Duke University, Durham, NC, United States of America; University of Portsmouth, UNITED KINGDOM

## Abstract

Understanding the behavior of others in a wide variety of circumstances requires an understanding of their psychological states. Humans’ nearest primate relatives, the great apes, understand many psychological states of others, for example, perceptions, goals, and desires. However, so far there is little evidence that they possess the key marker of advanced human social cognition: an understanding of false beliefs. Here we demonstrate that in a nonverbal (implicit) false-belief test which is passed by human 1-year-old infants, great apes as a group, including chimpanzees (*Pan troglodytes*), bonobos (*Pan paniscus*), and orangutans (*Pongo abelii*), distinguish between true and false beliefs in their helping behavior. Great apes thus may possess at least some basic understanding that an agent’s actions are based on her beliefs about reality. Hence, such understanding might not be the exclusive province of the human species.

## Introduction

Humans’ closest primate relatives, great apes, understand much about the psychological functioning of others. For example, they can identify the goals of others, as evidenced by their ability to distinguish between intentional and accidental actions and to identify what others want in order to help them [1, 2]. They can also identify what others perceive or do not perceive, as evidenced by their tendency to pursue food that a dominant cannot, as opposed to can, see (or even has not, as opposed to has, seen hidden in the immediate past) [3, 4]. And they can identify others’ desires, as evidenced by their ability to use others’ emotional expressions to infer their preferences for specific types of food and infer which type of food they have chosen [5].

But a key marker of advanced social cognition is an understanding of others’ false beliefs. For many years it was thought that great apes lacked this understanding: In a series of previous experimental studies, chimpanzees, bonobos, and orangutans showed no reliable evidence of passing false-belief tests [6–9]. In all of these studies, just as in the classic false-belief tests used with human children [10], in order to pass the test, the participants would have had to understand that an agent’s action was being directed in a particular way not because of reality but because of her beliefs about reality (e.g., the agent was looking for food in location A, not because it was there but because she believed it was there).

Recently, several new, less demanding false-belief tests have been developed. Although still based on the classic tests that young human children pass only at 4 to 5 years of age [11], these new tests are different in that they are passed by human infants at 1 to 2 years of age. For example, in a violation-of-expectation paradigm pioneered by Onishi and Baillargeon [12], 15-month-old infants witnessed an actor behaving either congruently or incongruently with her beliefs in true- and false-belief situations. For example, in the false-belief condition, the actor did not see an object being moved to a new location but nevertheless searched for it there (incongruently to her beliefs). Infants looked longer at this scene compared to when the actor looked for the object where she believed it to be. These findings are taken as evidence that even human infants implicitly expect others’ behavior to be driven by their beliefs. (For further studies of this type, some with even younger infants, see, e.g., [13–15], and for a review see [16]. See also [17] for a demonstration that rhesus monkeys fail the Onishi & Baillargeon test.)

Recently, Krupenye, Kano, Hirata, Call, and Tomasello [18] tested great apes using one of these implicit tests, the anticipatory-looking false-belief test developed by Southgate, Senju, and Csibra [19]. In this study, like the human 2-year-olds in [19], chimpanzees, bonobos, and orangutans anticipated the location where an actor would search for an object based on the actor’s false belief about the object’s location (see also [9] for mixed results in a false-belief study measuring anticipatory looking with chimpanzees and bonobos). However, in the Krupenye et al. study, apes could have passed the test simply by predicting that the actor would go to the last place he saw the object; thus, converging evidence is needed from tests that use different methods. Furthermore, even if apes were using an understanding of false beliefs in [18], it is crucial to show that they can go beyond predicting others’ behavior and also use their understanding of beliefs appropriately in real social interactions.

In the current study, we thus tested great apes using one of the infant paradigms that use active behavioral (as opposed to looking) measures. Buttelmann, Carpenter, and Tomasello [15] had 16- and 18-month-old human infants watch while an experimenter placed a toy into a box. In the false-belief condition the experimenter then left the room and infants watched as a research assistant moved the toy to a different box. The experimenter then returned and approached the box in which he had originally placed his toy and tried unsuccessfully to open it. Infants were then given the chance to help him. The majority of infants in this condition did not try to help him open the box he was struggling with, but instead helped him retrieve the toy from the other box. In contrast, in the true-belief condition, in which the experimenter stayed in the room and watched the transfer of the toy, infants behaved differently: Rather than going to get the toy, infants more often helped him to open the box he was struggling with. Infants apparently inferred that when the experimenter falsely believed that his toy was in the original box, his attempts to open that box must be directed at the (now moved) toy, whereas when he knew that the toy had been moved from the original box, his goal must have been to open that box for some other reason. Thus, by differing in their helping behavior between conditions, infants demonstrated their ability to attribute different goals to the experimenter based on his true vs. false beliefs. Here we used Buttelmann and colleagues’ [15] test to investigate whether great apes, too, can use an understanding of beliefs to attribute goals in a helping task.

## Study 1

### Method

#### Participants

A total of 34 great apes participated in this study: twenty-three chimpanzees (mean age = 20 years, 3 months; range = 5 years, 6 months to 48 years, 0 months; 16 females, seven males), five bonobos (mean age = 18 years, 11 months; range = 9 years, 0 months to 31 years, 2 months; three females, two males), and six orangutans (mean age = 19 years, 3 months; range = 4 years, 8 months to 33 years, 5 months; five females, one male). Twenty of these apes also participated in the Krupenye et al. [18] study, but the current studies were conducted before that one. The apes were housed in social groups at the Leipzig Zoo, Germany. Six additional chimpanzees (five females, one male) and two bonobos (both females) participated in the initial training but had to be dropped from the study because they failed the training criterion (see description below). One additional bonobo (male) was trained but had to be dropped because he could not be separated from the group for testing.

#### Ethics statement

The apes were housed socially, separated by species, in groups of at least six individuals at the Wolfgang Köhler Primate Research Center (WKPRC) in the Leipzig Zoo, Germany. Each group of apes has access to an indoor area (between 230 and 430 m^2^) and an outdoor area (between 1680 and 4000 m^2^) furnished with various climbing structures, shelter and natural vegetation. At night, the apes sleep in several series of cages (between 40 and 50 m^2^). In addition to experiments, the apes are provided with a special enrichment program, including various kinds of tools and foraging containers. Several times per day, the apes are fed a diet consisting primarily of vegetables, fruits, and cereals, with regular additions of eggs and meat (depending on the species). Test sessions took place in the participants’ sleeping enclosure (see above for sizes). The participants were used to being separated in adjacent enclosures from their group members for testing. They were not food deprived for testing, and water was available throughout all test periods. They were not distressed and were free to stop participating at any time. No animals were sacrificed, and German law on animal rights and ASAB/ABS guidelines [20] were followed throughout. An internal WKPRC ethics committee, consisting of Zoo Leipzig personnel and MPI-EVA academic research staff, approved the studies. IRB approval was not necessary because Germany requires no special permission for the use of animals in purely behavioral or observational studies (TierSchGes §7 and §8).

#### Materials and set-up

**Fig 1:**
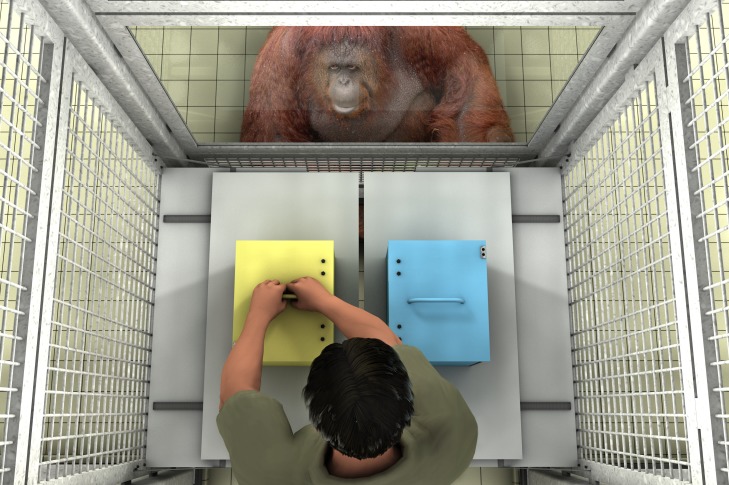
The set-up in Study 1. Right before the response phase, the experimenter tries to open the box which he believes contains his object (false-belief condition) or which he knows is empty (true-belief condition). In both conditions this is the same empty box; all that differs is the experimenter’s belief about what is in it.

Materials for this study were two plastic boxes (30 x 22.5 x 20 cm, one yellow, one blue; otherwise identical), each with a hinged lid, a handle on top, and a plexiglass front. The lids of the boxes could be locked and unlocked by moving a bolt horizontally on the front, see S1 Fig. When the lid was open, the locking mechanism was not visible from the top because it was covered (so the experimenter could not see it during the test). Both boxes were put on sliding tables opposite each other which were attached to the outside of participants’ enclosure, so that they could be moved into and out of the participants’ reach, see Fig 1. The plexiglass front was visible to participants when they moved to either side of the testing booth during the response period (i.e., not, in contrast, while being centered right before test, as in Fig 1) whereas the assistant and the experimenter could only see the top and the back side of the boxes, and thus not their contents. In one type of training trials (*Training on empty boxes*) these boxes were empty. In the other type of training trials (*Training on boxes containing an object*) a bunch of four keys was put into the boxes. At test, instead of the keys a novel object (a small orange box, 10cm x 7cm x 7cm, filled with stones that made noise when shaken) was put into the boxes.

#### Design and procedure

Apes participated in three rounds of testing, each of which consisted of training on empty boxes, training on boxes containing an object, and two test trials. The delay between the training on empty boxes and the training on boxes containing an object was one to four weeks in each round. The two test trials were run on two consecutive days immediately after the training on boxes containing an object. Participants were randomly assigned to one of the two conditions (see below) for all test trials (between-subjects design, *n* = 17 for each condition). The physical set-up was identical in all training and test trials (except for the object that was placed in the boxes). The training trials were identical for all participants independent of condition.

*Training on empty boxes*. These training trials were conducted to make sure that the participants were able and motivated to unlock empty boxes. At the beginning both boxes were empty and unlocked. The assistant sat on a stool centered behind the two boxes and called participants by name. While they watched, she first opened the lid of the box on the left. She then closed the lid and locked it by moving the bolt horizontally. To demonstrate that the box was locked she pulled on the handle on top of the lid (three short pulls) and subsequently pushed the box into participants’ reach. Participants could unlock the box by moving the bolt in the other direction. When participants unlocked the box, the assistant pulled back the box, opened the lid, and made a pleasant vocalisation (“Mm hm”). Participants were given a grape from the assistant’s chest pocket and the trial was over. This procedure was repeated four times for a total of five training trials on this box. The assistant then repeated the same procedure with the box on the right.

In the rare case that participants did not unlock the box within 10 seconds, the assistant pulled the box out of participants’ reach, pulled the handle again (three short pulls), and pushed the box back into participants’ reach. If necessary, this was repeated two more times, each after 10 seconds. If participants did not unlock the box in any of these four attempts the assistant pulled back the box, unlocked it, and opened the lid with a neutral face, and this training trial ended. The assistant then proceeded with the next training trial. In order to pass this training session, participants needed to unlock a box at least once in the ten training trials; otherwise they were excluded from the study.

*Training on boxes containing an object*. The procedure of these training trials was similar to that of the training on empty boxes except that the assistant now put a bunch of keys into the box each time before she closed the lid. After participants unlocked the box, she opened the lid, took the keys out, made a pleasant vocalisation (“Mm hm”), gave participants a grape, and proceeded with the next trial. Participants received three consecutive trials on each side. After this training all participants had unlocked a maximum of ten empty boxes and six boxes that contained an object (and a minimum of one each).

*Test*. The basic procedure of the test followed that of Buttelmann, Carpenter, and Tomasello [15] with human infants. First, participants received two more training trials with boxes containing an object on each side, as in the Buttelmann et al. study (and to give them an equal number of training trials with and without objects in the box). After this, the assistant ensured that both boxes were unlocked and left the scene (i.e., went back behind the stool and turned her back). The experimenter (E) entered the room, sat down centered behind the two boxes (with the side of the yellow box counterbalanced across participants), and showed the novel object (the small orange box) to participants. E played with it for a little while to ensure that the object was equally enhanced for all participants. Then E opened the lid of one box (counterbalanced), put the object into it, said and waved goodbye to the participants and the assistant, and left the room. For all of these actions, E commented to himself about what he was doing to keep the procedure similar to the original test with human infants. What followed differed between conditions:

In the *true-belief* condition the assistant returned and stood centered behind the two boxes. E also returned and stood behind the assistant so that he could witness what happened next. The assistant called both participants and E by their names and commented on all of her subsequent actions. She opened the lid of the box that contained the object, took it out of the box, closed the lid, and locked the box. Then she opened the lid of the other box, put the object in, closed the lid, and locked that box. E expressed that he was paying attention to the whole process by saying “Aha” whenever a lid was lifted and the object was taken out of or put into a box. He always turned his back when the assistant locked a box so that later, at test, it would be plausible that he did not know how a locked box could be unlocked. After that the assistant centered participants with food (e.g., raisins, grapes, or food pellets) and left the scene.

The procedure of the *false-belief* condition was similar to that of the true-belief condition with the exception that E stayed outside until the assistant had transferred the object to the other box and, therefore, did not witness the switch. In this condition, the assistant behaved “sneakily,” and looked furtively at the door to the room whenever a lid was opened and the object was taken out of or put into a box to ensure that E was not watching. Then the assistant centered participants, E re-entered the room, and the assistant left the scene. In both conditions the assistant ensured that participants witnessed her actions throughout the procedure and paused if participants became distracted.

What followed was identical in both conditions: E stood behind the empty box (the one he had originally put the object in) and unsuccessfully tried to open it by pulling the handle (three short pulls), showing effort. He then showed a helpless gesture (i.e., raised his shoulders, held up both hands, palms up, made an uncertain facial expression, and said uncertainly, ‘‘Hm”), still leaning towards the empty box. He then sat down on the stool, called participants by name (“[Name], look!”), pushed both boxes into participants’ reach simultaneously, and bent his head down, centered between the two boxes, so that he could not inadvertently provide any gaze or facial cues for participants. As soon as the boxes were in participants’ reach, it was their turn to unlock a box. If participants unlocked a box, E pulled back both boxes, opened the lid of the box participants had unlocked, and looked into it, saying something like “That is how the box opens” or “Oh, the object is in here now.” He then left the room without ever touching the object in the box. Finally, independent of which box was unlocked by participants, the assistant returned and rewarded them with a grape from her pocket in the center. Participants received another two training trials and another test trial on the following day. Since the whole training and testing procedure was repeated twice, participants received three rounds of testing, each consisting of the training sessions and two days of testing (i.e., each with two training trials and a test trial), for a total of six test trials.

During test trials, in order to ensure that participants 1) watched E’s attempt to open a box and 2) started in their choice of which box to unlock from a centered position, we required that they be centered whenever E was pulling on the handle of the empty box and when he pushed both boxes into participants’ reach. If participants left the center area after the pulling, E stopped and stepped back. Participants were re-centered by the assistant, and E pulled the handle again. If participants did not choose a box within ten seconds, E pulled back the boxes, looked at the participants and called them by name, showed the helpless gesture, and pushed both boxes back into the participants’ reach. This was repeated each time ten seconds passed without a choice by participants for a maximum of seven such ten-second periods. In the rare cases that participants did not choose at all, the trial was counted as “no choice” (blank, see S1 Table). If participants chose a box (i.e., they touched the bolt) but did not successfully unlock the lid, E pulled both boxes back and tried to open the chosen box. Since the box was still locked (e.g., the bolt was not moved far enough by the participants), E showed the helpless gesture again, looking at the participants, and pushed the boxes back into the participants’ reach to give them another chance to be successful. Importantly, E did not look at either box after he had pulled the handle of the empty box until the trial was over. The only exception was when participants chose a box without successfully unlocking it, and E subsequently tried to open it (see above). Note that at this point of the response period participants had already chosen a box by touching the locking mechanism, which was the main measure (as in Buttelmann et al.’s [15] study).

The response period, starting from when E first pulled on the box, was 90 seconds (as in [15]). During this time, participants chose a box in 88.2% of the trials. If participants could not be centered at all and E could not pull on the empty box the trial was stopped after 3.5 minutes and re-conducted either later on the same day or the next day. If centering was also impossible during the second attempt or if the second attempt was not possible because of the testing schedule (i.e., no more testing time was available for that round) the trial was counted as “no choice” (blank).

We coded which box participants touched first. The touch had to be directed at the locking mechanism, and participants did not need to unlock it, although they did do this in most cases. Thus, only first response data for each trial were analyzed. To assess inter-rater reliability, a naïve coder independently coded 100% of trials blind to condition. Perfect agreement was achieved, Cohen’s Kappa = 1.00. All *p* values reported are two-tailed.

### Results and discussion

Like human infants in [15], apes correctly unlocked the box containing the object significantly more often in the false-belief than in the true-belief condition (means: 76.5% vs. 53.1% of six trials, respectively; Mann-Whitney *U* = 74.50, *N*_*false*_ = *N*_*true*_ = 17, *Z* = -2.44, *p* = .015, *r* = .42). They unlocked this box significantly more often than chance in the false-belief condition (Wilcoxon test, *T*^+^ = 129.00, *N* = 16 (1 tie), *p* = .001, *r* = .78). In the true-belief condition, in contrast, they unlocked both boxes equally often (Wilcoxon test, *T*^+^ = 38.50, *N* = 11 (6 ties), *p* = .493, *r* = .12). Although participants were not differentially rewarded after each trial, we checked whether they might have learned the correct answer over time by comparing the number of successful participants across trials. No differences could be found (Cochran’s *Q* test, *χ*^2^(5) = 4.59, *p* = .467). See the SI, and S1 Table in particular, for the results separately by species, trials, and testing session.

Apes thus behaved differently in the two conditions, correctly unlocking the “other” box (the one the experimenter was not tugging at) significantly more often in the false-belief condition than in the true-belief condition. Given that at test everything except what the experimenter believed about the location of the toy was identical in both conditions, apes had to go beyond the observable facts of the situation to figure out the experimenter’s goal. However, before being able to conclude that they were using an understanding of beliefs to do this, we first need to rule out a possible alternative interpretation of the results that is related to apes’ well-documented understanding of others’ knowledge/ignorance [7–9]. That is, in the false-belief condition, rather than understanding that the experimenter had a false belief about the current location of the object, apes might have understood that he was ignorant about the current location of the object and responded based on a rule such as ‘whenever someone is ignorant about where his object is, he will be looking for it.’ Although these two different levels of understanding (false belief versus ignorance) would result in exactly the same response behavior in the current task, the underlying cognitive representations would be different [19].

To test this possible alternative explanation, in Study 2 we compared apes’ performance in a replication of the false-belief condition with their response behavior in an ignorance control condition. Whereas in the false-belief condition the experimenter had a specific belief about the location of the object (i.e., that it was in the box he tried to open at test), in the ignorance condition he had no idea where the object might now be. If participants were using a rule based on an understanding of the experimenter’s ignorance rather than false belief in the false-belief condition, they should behave similarly in these two conditions, whereas if they were using an understanding of false belief in one and ignorance in the other they should behave differently. That is, in the ignorance condition, in contrast to the false-belief condition, they should perform at chance, like 18- and 30-month-old human children in false belief studies that include this type of control condition [21–23].

## Study 2

### Method

#### Participants

Participants were 29 of the apes who had participated in Study 1: nineteen chimpanzees (mean age = 20 years, 1 month; range = 6 years, 8 months to 40 years, 4 months; 13 females, six males), four bonobos (mean age = 16 years, 2 months; range = 9 years, 4 months to 23 years, 10 months; three females, one male), and six orangutans (mean age = 19 years, 6 months; range = 5 years, 0 months to 33 years, 9 months; five females, one male). Two of the chimpanzees (one female, one male) from Study 1 were no longer available for testing, and three of the other apes who had participated in Study 1 (two female chimpanzees, one male bonobo) were trained but had to be dropped from this study because they did not open a box during training (*n* = 1), or else could not be centered during testing and did not open a box at test (*n* = 2). Individuals were randomly assigned to either the ignorance condition (*n* = 15) or a new, matching false-belief condition (*n* = 14), with the stipulation that approximately half of the participants in each condition had been tested in the true-belief and half had been tested in the false-belief condition of Study 1.

#### Materials and set-up

Materials for this study were two new opaque plastic boxes (30 x 22.5 x 20 cm, one green, one orange), each with a hinged lid and a handle on top and a plexiglass front. The lids of the boxes could be locked by turning a bolt on the front clockwise, see S2 Fig. Both boxes were positioned in the same way as in Study 1. The same bunch of keys as in Study 1 was put into the boxes during the training trials with boxes containing an object. At test, a novel object (a small round box, 10 cm in diameter with six small conical boxes attached to it, filled with stones that made noise when shaken) was put into the boxes.

#### Design and procedure

Study 2 was run two to six months after Study 1 was finished. The design and set-up of the training was identical to that of Study 1 with the exception that, due to an observable decrease in participants’ tendency to open boxes in the training sessions, in this study training trials were repeated once if participants did not at least touch the bolt. The basic procedure of the test followed that of Study 1. What differed was that in both conditions, after E had played with the novel object, the assistant asked (verbally and by presenting an open hand to E) if she could have the object, and E handed it to her. Then E opened the lid of one box (to keep the procedure comparable in the ignorance and false-belief conditions). Which box E opened was counterbalanced across trials. What followed differed between conditions:

In the *ignorance* condition, E leaned over the opened box and said, “The box is made of wood” twice, alternating gaze between the box and participants. He then said and waved goodbye to the participants and the assistant (who was still holding the object) and left the room. The assistant stood centered behind the two boxes and called the participants by name. She put the object into the opened box. After two seconds she took it out of the box, closed the lid, and locked the box. Then she opened the lid of the other box, put the object in, closed the lid, and locked that box. After that, the assistant centered participants with food (e.g., raisins, grapes, or food pellets) and left the scene. Then E returned, ignorant (i.e., having no specific belief) about where the object was.

The procedure of the matching *false-belief* condition was similar to that of the ignorance condition with the exception that after E had opened one box, E watched as the assistant put the object into it. E then leaned over the box containing the object and said, “The object is in here” twice, alternating gaze between the box and participants. He then said and waved goodbye to the participants and the assistant and left the room. While he was away, the assistant sneakily switched the location of the object and left the scene before E returned.

The procedure of the test was identical in both conditions and identical to Study 1. Coding criteria and counterbalancing were also identical to Study 1. Participants chose a box within the response period in 92.0% of the trials. To assess inter-rater reliability, a naïve coder independently coded 100% of trials blind to condition. Excellent agreement was achieved, Cohen’s Kappa = .99.

### Results and discussion

Participants’ performance differed significantly between the false-belief and the ignorance condition (Mann-Whitney *U* = 55.00, *Z* = -2.21, *p* = .027, *r* = .41). In the false-belief condition, again, apes correctly unlocked the box containing the object significantly more often than chance (mean: 72.0% of their six trials; Wilcoxon test, *T*^+^ = 74.00, *N* = 12 (2 ties), *p* = .006, *r* = .74), thus replicating the findings of the false-belief condition in Study 1. In contrast, as expected, in the ignorance condition participants chose randomly between the empty box and the box containing the object (means: 49.9% vs. 50.1% of six trials, respectively; Wilcoxon test, *T*^+^ = 29.00, *N* = 10 (5 ties), *p* = .877, *r* = .04). Although participants were not differentially rewarded after each trial, we checked whether they might have learned the correct answer over time. They did not, as performance was constant across trials (Cochran’s *Q* test, *χ*^2^(5) = 1.04, *p* = .959). See S1 Table for performance on individual trials and the SI for additional results. The finding that apes behaved differently in the false-belief and ignorance conditions suggests that they used an understanding of the experimenter’s false belief, rather than a simpler rule based on his ignorance, to decide how to help him in the false-belief conditions of these studies (see, e.g., Kaminski et al., 2008, for further support for the idea that apes do not use similar behavioral rules to pass these kinds of tests).

## General discussion

Using one of the new interactive behavioral infant false-belief tests [15] with apes, we found evidence that apes may have a basic understanding of others’ false beliefs. In Study 1, they responded differently in the true- and false-belief conditions. In Study 2, which replicated the false-belief condition results from Study 1, they behaved differently in the false-belief condition and an ignorance control condition designed to rule out an explanation based on a simpler rule involving ignorance rather than beliefs. These findings support the recent findings of Krupenye et al. [18], and extend them by 1) using a method in which some of the criticisms leveled at the anticipatory-looking procedure do not apply, and 2) showing that in addition to being able to anticipate others’ behavior based on their beliefs, apes can use their understanding of beliefs in real social interactions to work out what others are trying to accomplish and help them appropriately.

In both the false-belief and the ignorance condition, apes behaved exactly as they should have. In the false-belief condition in both studies, they chose the box with the object in it at above-chance levels, apparently assuming that since the experimenter tried to open the box that he believed contained his object, obtaining the object was his goal. In the ignorance condition, in contrast, apes chose the box with the object at chance level. This was the expected response in this condition because the experimenter did not have any specific belief about where his object might be: The last time he saw it was in the assistant’s hands when he left the room, and she no longer had it when he returned. At test, when he pulled on the box, participants had to figure out what he wanted, with little to go on (even if they considered what he knew or believed): Did he want the object and believe that maybe the assistant had put it in there? Or did he want to open the box again? Both answers were equally plausible given what had happened up to this point, and this is likely why half of the participants chose each of these options. Note that participants had to infer what the experimenter wanted when he returned and tried to open the empty box in the false-belief condition too, it is just that there the likely answer was much more straightforward, as long as they were considering his beliefs: Since he believed that the box he tried to open contained his object, he must want his object.

In the true-belief condition, however, the results were less clear-cut because apes chose both boxes equally often, instead of choosing more often the box without the object. When the experimenter tugged on the box that he knew to be empty, to adult humans (and to 18-month-old infants [15]) the most natural inference is that he must want to open the empty box for some reason–he cannot want the toy because he knows where it is and he is not trying to open that box. Several factors may have conspired to make that inference too difficult for apes, and possibly 16-month-olds [15]. For example, generally speaking, making an inference based on the absence of evidence is more difficult than one based on the presence of evidence (see [24] for a review), and, more specifically, inferring someone else’s goal in the absence of a tangible target is difficult. Although several studies have shown that apes are sensitive to the goals of others [e.g., 25–27], in those studies the experimenter always directed her actions towards an object or end state of that object. The goal of opening an empty box in the current true-belief condition might have been difficult to interpret for some of our participants (i.e., those who chose the box with the object in it). Support for this idea comes from imitation studies showing that actions aimed at a target are easier for apes to copy than those without a specific target [28–30] and from neurophysiological studies showing that mirror neurons in the monkey brain are activated specifically by object-oriented actions compared to actions that are not directed towards a visible or non-visible object [31]. Note that 15-month-old human infants in Onishi and Baillargeon’s [12] true-belief condition also expected the actor to approach the box that she knew contained an object instead of an empty box. Thus, it might be difficult to infer the goal behind opening an empty box. (This argumentation does not conflict with the finding in the current false-belief conditions, in which the experimenter also tugged on an empty box, because there his goal was the object he represented in that box.) However, another possibility is that participants were able to infer a goal; it is just that half of them inferred that the goal was to open the empty box and half, for some reason, inferred that it was to get the object. Additional research is needed to determine the most likely explanation for apes’ response in this condition.

In any case, it is worth noting that the 16-month-olds in [15] showed exactly the same pattern of results in the true- and false-belief conditions in this test: They too showed a significant difference between the true- and false-belief condition, chose the box with object more often than chance in the false-belief condition, and chose it at chance level in the true-belief condition. At the time, Buttelmann and colleagues interpreted their results cautiously, but since then at least four published studies (which include a variety of appropriate control conditions) have now reported positive findings with infants aged even younger than 16 months [12, 32–34]. We interpret the current results with caution too; however, as noted above, a recent study using a different implicit false-belief test with apes [18] provides converging evidence for our conclusion that great apes may indeed have a basic understanding of others’ false beliefs.

One might wonder why apes performed better in these two tests than in previous false-belief tests. One key difference between the classic and the infant false-belief tests is that in the classic tests, participants must reason about the actor’s beliefs in order to explicitly predict her behavior, whereas in the infant tests no prediction, or only an implicit prediction (as reflected by anticipatory looking [19, 35, 36]), is needed. That the current procedure required no prediction cannot be the only explanation for why apes passed this test but not previous false-belief tests, however, as in the previous tests with apes [6–9] there was also no prediction involved.

Another possible factor for apes’ success in [18] and the current study could be that in all other previous studies apes needed to discern the other’s beliefs in order to get food for themselves, which might have distracted them or required higher levels of inhibitory control from them. Here, in contrast, apes’ receipt of the reward did not depend directly on their performance at test, since they were rewarded regardless of performance in each trial. Thus, perhaps they were less distracted by the reward in these studies and so could make better use of their sophisticated social-cognitive skills. Finally, one reason apes may have succeeded in the current study is that here participants were required to infer the human’s goal and help him achieve it. Reading others’ goals is something that apes are quite skillful at in other contexts [25–27, 37]. However, it is important to note that in the current study, determining the actor’s goal differentially in the different conditions in addition required some inference about the different underlying beliefs of the actor.

The current results provide another indication that great apes might have some level of false-belief understanding, and they are the first to demonstrate that apes are able to use this understanding in their social interactions. If supported by further research, the apparent difference between great ape and human social cognition would thus lie not in their basic capacity to “read” other minds, but elsewhere.

## Supporting information

S1 Fig The blue apparatus used in Study 1 and an illustration of how it could be opened. (A) The locked box with object as seen from the participants’ perspective. (B) The unlocked box (i.e., bolt slid to the left). (C) The apparatus with the lid open.
(TIF)

S2 Fig The orange apparatus used in Study 2 and an illustration of how it could be opened. (A) The locked box with object as seen from the participants’ perspective. (B) The unlocked box (i.e., bolt turned counterclockwise). (C) The apparatus with the lid open.
(TIF)

S1 Table The performance of the individual participants in each trial in both studies. Abbreviations in the column “Condition in study” represent the false-belief (FB) and true-belief (TB) conditions of Study 1 and the false-belief (FB2) and ignorance (I) conditions of Study 2. Values in the column “Response” represent a choice of the empty box (“0”) or the box with object (“1”); blank fields reflect missing values (e.g., trials with no choice).
(JPG)

S1 File Supporting information for Studies 1 and 2. Here we report additional results.
(PDF)
