# Supplementary figures and images for: Great apes distinguish true from false beliefs in an interactive helping task
Source: PLoS One. 2017 Apr 5;12(4):e0173793. doi: 10.1371/journal.pone.0173793 (PMC5381863; doi:10.1371/journal.pone.0173793)

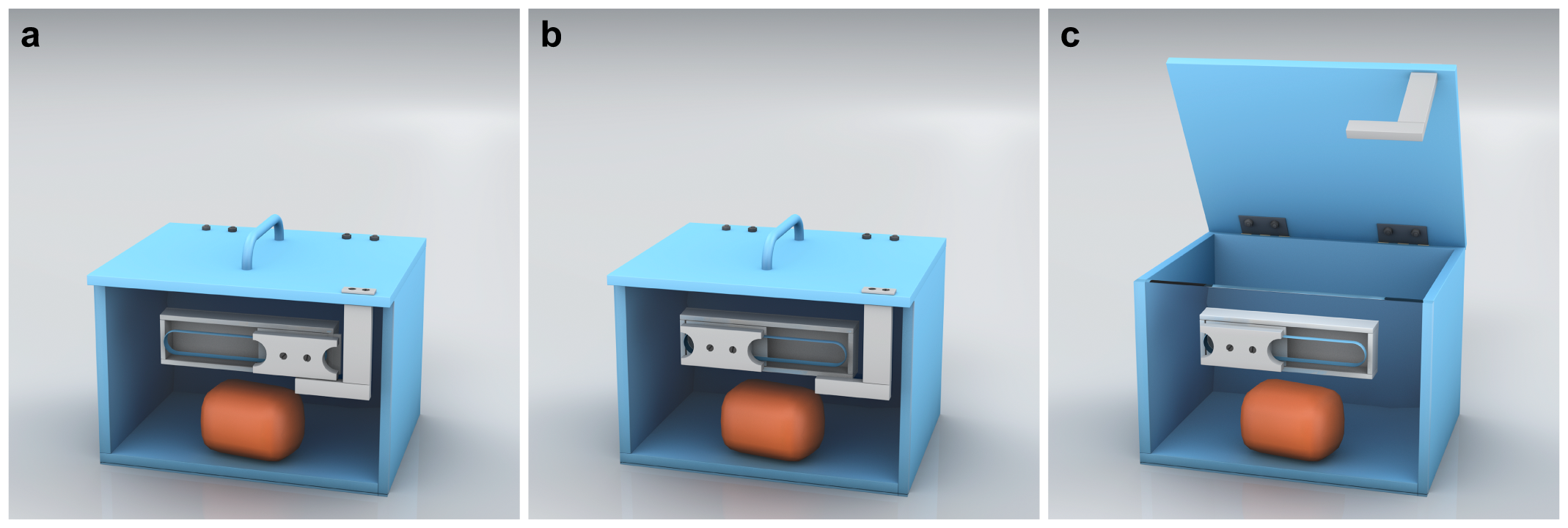

Supplement: S1 Fig — (A) The locked box with object as seen from the participants’ perspective. (B) The unlocked box (i.e., bolt slid to the left). (C) The apparatus with the lid open. (TIF) [file pone.0173793.s001.tif]

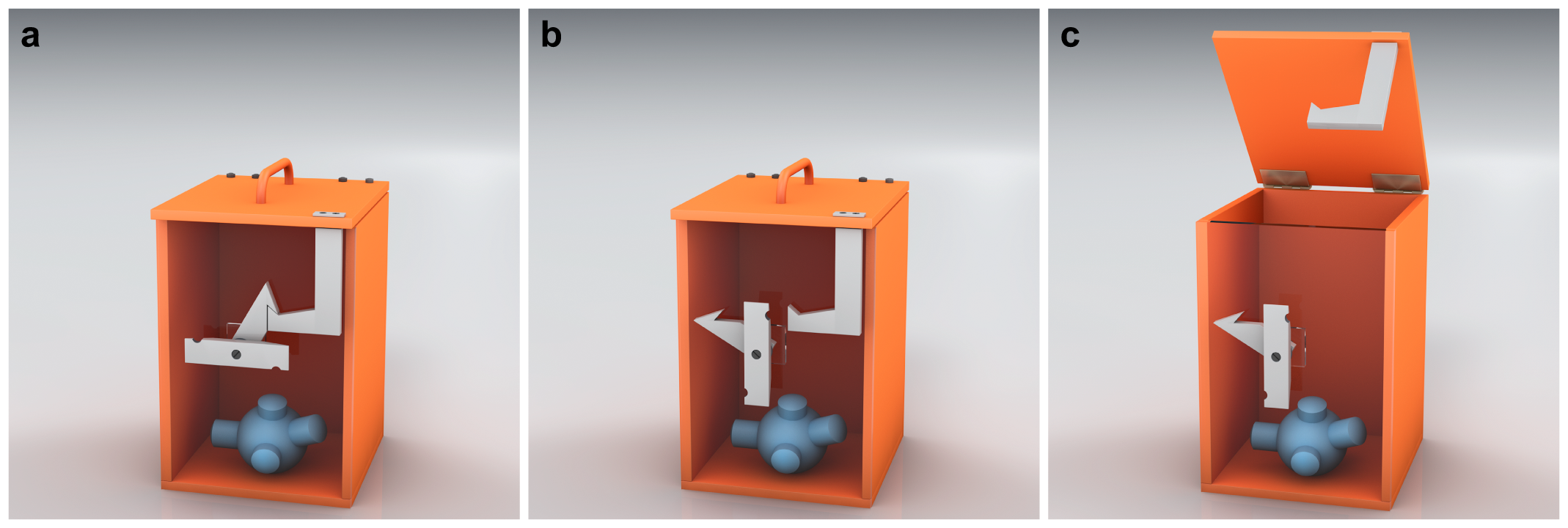

Supplement: S2 Fig — (A) The locked box with object as seen from the participants’ perspective. (B) The unlocked box (i.e., bolt turned counterclockwise). (C) The apparatus with the lid open. (TIF) [file pone.0173793.s002.tif]

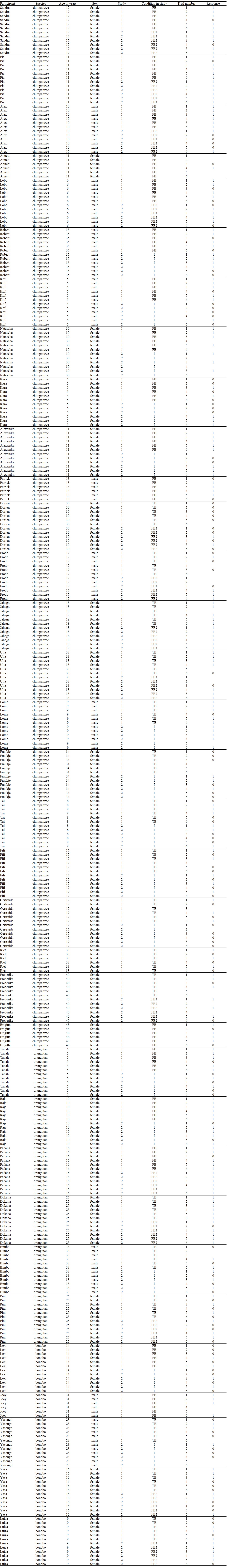

Supplement: S1 Table — Abbreviations in the column “Condition in study” represent the false-belief (FB) and true-belief (TB) conditions of Study 1 and the false-belief (FB2) and ignorance (I) conditions of Study 2. Values in the column “Response” represent a choice of the empty box (“0”) or the box with object (“1”); blank fields reflect missing values (e.g., trials with no choice). (JPG) [file pone.0173793.s003.jpg]
