# Supplementary material for: Great apes distinguish true from false beliefs in an interactive helping task
Source: PLoS One. 2017 Apr 5;12(4):e0173793. doi: 10.1371/journal.pone.0173793 (PMC5381863; doi:10.1371/journal.pone.0173793)
Supplement: S1 File — Here we report additional results. (PDF) [file pone.0173793.s004.pdf]

Great apes distinguish true from false beliefs in an interactive helping task

Supporting Information

David Buttelmann, Frances Buttelmann, Malinda Carpenter, Josep Call, Michael Tomasello

Supporting Information for Study 1

Additional Results

Training

Participants unlocked the boxes in empty box training trials in a minimum of 73.3% and a maximum of 100% of trials, and in box with object training trials in a minimum of 33.3% and a maximum of 100% of trials across the whole study.

Test

Chimpanzees unlocked the box containing the object in 75.0% of trials in the false-belief and in 51.2% of trials in the true-belief condition, bonobos did so in 66.7% and 46.7% of trials, and orangutans opened this box in 88.9% and 66.7% of trials, respectively. There were no differences between species with respect to correct performance (assuming the box with object to be correct in the false-belief condition and the empty box to be correct in the true-belief condition) [Kruskall-Wallis test, $\chi^2(2) = .22$, $p = .896$]. Participants showed the same pattern of results in each of the three rounds of testing (means: 1st round: 78.1% in false belief vs. 55.9% in true belief; 2nd round: 73.5% vs. 64.7%; 3rd round: 81.3% vs. 36.7%, respectively). In the 1st round this difference between conditions approached significance [Mann-Whitney *U* test, $U = 91.5$, $N_{false} = 16$,

N_{true} = 17, Z = -1.76, p = .079], and in the 3rd round it was significant [U = 48.0, N_{false} = 16, N_{true} = 15, Z = -3.05, p = .002]. There were no effects of participants' sex or age.

Supporting Information for Study 2

Additional Results

Training

Participants unlocked the boxes in empty box training trials in a minimum of 63.3% and a maximum of 100% of trials, and in box with object training trials in a minimum of 76.8% and a maximum of 100% of trials.

Test

Chimpanzees unlocked the box containing the object in 71.3% of trials in the false-belief and in 54.3% of trials in the ignorance condition, bonobos did so in 66.7% and 29.2% of trials, and orangutans opened this box in 77.8% and 50.0% of trials, respectively. There were no differences between species with respect to correct performance [Kruskall-Wallis test, $\chi^2(2)$ = .52, p = .772]. Subjects showed the same pattern of results in each of the three rounds of testing (means: 1st round: 75.0% in false belief vs. 50.0% in ignorance; 2nd round: 67.9% vs. 50.0%; 3rd round: 75.0% vs. 50.0%, respectively). However, only in the 3rd round did this difference between conditions approach significance [Mann-Whitney U test, U = 63.0, N_{false} = $N_{ignorance}$ = 14, Z = -1.76, p = .079]. There were no effects of sex or age.

The performance of participants who had previously been tested in the true-belief and false-belief conditions in Study 1 did not differ in this study – both showed the correct pattern of responding. The apes previously tested in the false-belief condition opened the box with the object in 76.7% of trials in the false-belief and in 53.3% of trials in the ignorance condition; the means for the participants previously tested in the true-belief condition were 69.4% and 46.4% of trials, respectively.
